# Airway Inflammation Responses to Walking Roadside and Park Routes After School: A Real‐World Crossover Study

**DOI:** 10.1002/ejsc.12280

**Published:** 2025-04-30

**Authors:** Scarlett Moloney, Gavin Devereux

**Affiliations:** ^1^ School of Allied Health Sciences University of Suffolk Ipswich UK; ^2^ Cambridge Centre for Sport and Exercise Sciences School of Psychology and Sport Science Anglia Ruskin University Cambridge UK; ^3^ Institute of Health and Wellbeing Research University of Suffolk Ipswich UK

**Keywords:** active travel, air quality, airway inflammation, physical activity

## Abstract

Active travel to and from school is encouraged as a form of daily exercise. However, a significant proportion of children's pollution exposure has been attributed to this time due to road traffic volume. We investigated fractional exhaled nitric oxide (FeNO) responses in children walking roadside and park routes after school. 18 children (9.6 ± 1.0 years) completed six 30 min walks immediately after a school day (three in each environment). FeNO was measured before and after, with pollution concentrations measured during each walk. FeNO was reduced following roadside (20.87 ± 17.14 vs. 18.96 ± 15.63 ppb and *p* = 0.006) and park walks (19.13 ± 2.22 vs. 16.60 ± 2.74 ppb and *p* < 0.001). The reductions were not different between the two environments. The ICC for all pre‐walk FeNO was good (0.882 95% CI: 0.792, 0.947). Measured PM_2.5_ (5.9 ± 2.2 vs. 6.5 ± 2.6 μg/m^3^), PM_10_ (14.9 ± 11.9 vs. 14.8 ± 8.1 μg/m^3^) and VOCs (132 ± 91 vs. 80 ± 50 ppb) were not different between roadside and park conditions, respectively. Modest reductions in FeNO after walking suggest a normal response to light to moderate intensity exercise. The similar responses for roadside and park environments suggest that the benefits of active travel outweigh potential risk of pollution exposure where pollution concentrations are below current World Health Organisation thresholds. FeNO can also be reliably measured at the end of a school day with little direct control of behaviour in the hours before measurement.

**Summary**

- Fine (PM_2.5_) and coarse (PM_10_) particulate matter concentrations were equal during 30 min walks after school along busy roadside and park walking routes.
- PM_2.5_ and PM_10_ concentrations were just above and equal to annual thresholds set by the 2021 World Health Organisation Air Quality Guidelines, respectively. Both were below the 24 h thresholds from the same guidelines.
- Equal reductions in fractional exhaled nitric oxide (−1.9 ppb) occurred after walking both routes, suggesting a typical acute airway response to exercise in children. More research is needed to understand the long‐term health implications of pollution exposure during active travel to and from school.

## 1 Introduction

Air pollution exposure is associated with up to 1 in 5 deaths globally, making it the most prevalent environmental threat to public health (World Health Organisation 2021). For children especially, the adverse health effects associated with air pollution exposure can be significant, by impairing lung development, provoking exacerbations in respiratory symptoms and increasing the onset of asthma (Gauderman et al. 2007; Han et al. 2021).

Despite the health benefits associated with physical activity, a significant proportion of children's daily pollution dose has been attributed to their commute to and from school due to the peaks in road traffic associated with these times of day (Rivas et al. 2016). Children will likely experience an increased volume of inhaled pollutants due to an increased metabolic rate, breathing frequency and tidal volume in response to physical activity. This poses the question whether actively commuting to and from school during times of peak air pollutant concentration could amplify adverse health effects associated with pollutant exposure?

Various strategies have been used to promote active travel to and from school as an effective method of increasing children's daily physical activity to contribute towards the 60 min of exercise recommended per day by the World Health Organisation (WHO). For children living in suburban and urban areas, it is thought that more than 50% of the time they spend being physically active is attributed to their active commute to and from school (Rainham et al. 2012). Approximately 49% of children in four European countries walk both to and from school each day, with up to a further 22% cycling both ways in Norway (Haug et al. 2021). In the United Kingdom, over 80% of children walk to and from school when the distance is 1.6 km or less (Department for Transport 2024). Alongside the associated health benefits and reduction in all‐cause mortality (Kelly et al. 2014), increased use of active travel instead of commuting by car could also provide benefits to the environment, considering the probable reduction in traffic density and traffic related air pollution concentrations.

Compared to adults, children are at an increased risk to the adverse health effects of air pollution because of physiological and behavioural differences. For example, children have a higher breathing frequency and inhale a higher air pollution dose relative to body mass, and they also spend more time being physically active outside compared to adults (Adams and Requia 2017; Rivas et al. 2018). Avoiding walking along a main road on the commute to and from school is encouraged where possible, given the greater distance from mainstream traffic and the anticipated lower exposure to traffic related air pollution (Ahmed et al. 2020). Although these assumptions appear logical, there is little research that directly compares the acute effects of walking roadside and non‐roadside routes on important health parameters, such as airway inflammation, that can be immediately impacted by air pollution exposure (Chen et al. 2020).

Fractional exhaled nitric oxide (FeNO) is a non‐invasive biomarker of eosinophilic airway inflammation (Smith et al. 2004) and has been measured in several studies to investigate the airway response to air pollution exposure (Chen et al. 2020). FeNO is a biomarker of airway inflammation widely used as a clinical tool to assess t‐helper 2 cell related asthma and airway inflammation (Bjermer et al. 2014). Unlike other biomarkers of airway inflammation that are measured via venous blood samples, FeNO is measured using a non‐invasive test that can be used in different settings. This, along with FeNO being a valid tool to assess the airway inflammatory response to air pollution exposure (Annesi‐Maesano and Dinh‐Xuan 2016), means measuring FeNO in this study was the most appropriate and practical choice to facilitate field testing. FeNO has been widely used to assess the airway inflammation response to air pollution in children (Berhane et al. 2011; Czubaj‐Kowal et al. 2021; Flamant‐Hulin et al. 2009; Mohd Isa, Jalaludin, Mohd Elias, Mohamed, Hashim, and Hashim 2022).

The results of existing research are conflicting. For example, a panel study over two months observed increases in FeNO in non‐asthmatic children 24 h after exposure to increased PM_10_ (Carlsen et al. 2016). Similarly, Mohd Isa and colleagues found PM_2.5_ and nitrogen dioxide (NO_2_) to be positively associated with FeNO (Mohd Isa, Jalaludin, Mohd Elias, Mohamed, Hashim, and Hashim 2022). However, others found no association between ambient air pollution and FeNO (Altuğ et al. 2014). This is perhaps influenced by the limitations of a cross‐sectional study design and/or reporting air pollution as a weekly rather than daily average concentration (Altuğ et al. 2014). Considering FeNO measurements are sensitive to transient increases in air pollution exposure (Chen et al. 2020), if air pollution is not measured as a daily average, or preferably even with live data capture using personal air pollution sensors, it may be more difficult to detect the FeNO response to varying air pollution concentrations.

There are few, if any, real‐world before‐after (pre‐post) studies that aim to investigate the effect of air pollution exposure captured by live data on acute changes in FeNO during the active commute to and/or from school. Considering actively commuting to and from school is promoted and the concerns about air pollution exposure to children's health, it is important to understand the combined impact of air pollution and walking on factors such as airway inflammation. It is also of interest to explore the potential efficacy of walking through a park, green space or non‐roadside route within a busy town or city as a feasible strategy to reduce exposure to air pollution. Therefore, the primary aim of this study is to investigate the airway inflammation response, measured by FeNO, to walking for 30 min after school along both roadside and park routes.

The secondary aim of this study is to evaluate the feasibility of conducting a real‐world before‐after (pre‐post) study with primary school children that fits around the school day. This includes the reliability of FeNO measurements at the end of the school day with little direct control of the children's behaviours the in hours prior to measurement.

## 2 Materials and Methods

*Participants and Ethics:* 18 school children (11 female, 7 male; 9.6 ± 1.0 years of age) completed all study procedures, following informed consent being provided by the child and their parent or legal guardian. Inclusion criteria were that the children must ordinarily walk to and from school at least three times per week. The opportunity to participate was advertised using the school's online communication software, IRIS Parentmail, whereby all parents/guardians received an email with links to further study information. Children in school years 4, 5 and 6 were invited to participate, which comprises an age range of 8–11 years. None of the children/parents reported a diagnosis of asthma, exercise‐induced bronchoconstriction or any other respiratory condition throughout the duration of study. The study was approved by the University of Suffolk Research Ethics Committee (RETH(S)22/027).

### 2.1 Study Design and Procedures

**FIGURE 1:**
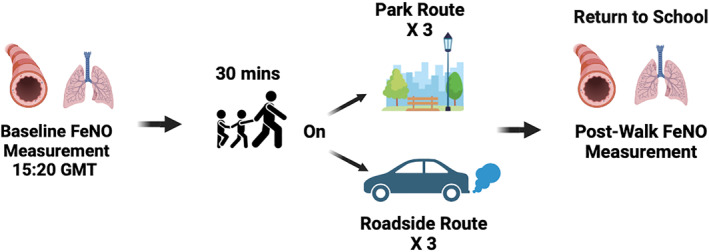
Schematic of the data collection session structure and study design.

The study adopted a repeated measures real‐world crossover design, whereby all children completed six 30 min walks immediately after the end of the school day, beginning at 15:20 h ± 5 min GMT. Both routes started and finished at the school. Each child took part in one session per week for 6 weeks. The data collection period began on 21st February 2023 and ended on the 31st of March 2023 with data collection taking place on 22 days in total. Some of the children required a catch‐up session during the week of 20th to 25th April 2023, after the school Easter break, due to absence from school during the February and March sessions. Three walks were completed along a busy roadside, and three walks were completed in a park setting adjacent to the school; the order of the sessions were counterbalanced. A study schema is presented with Figure 1.

The end of the school day was used rather than time before school as (i) all children were conveniently located in one place at the school, (ii) the children will have experienced similar physical activities and other behaviours during the school day and (iii) there would be a minimum of 2.25 h since the last food was consumed during the school lunch period, meeting the requirements of guideline recommendations for testing, as food intake can affect FeNO levels (Matsunaga et al. 2021).

#### 2.1.1 Walking Routes

**FIGURE 2:**
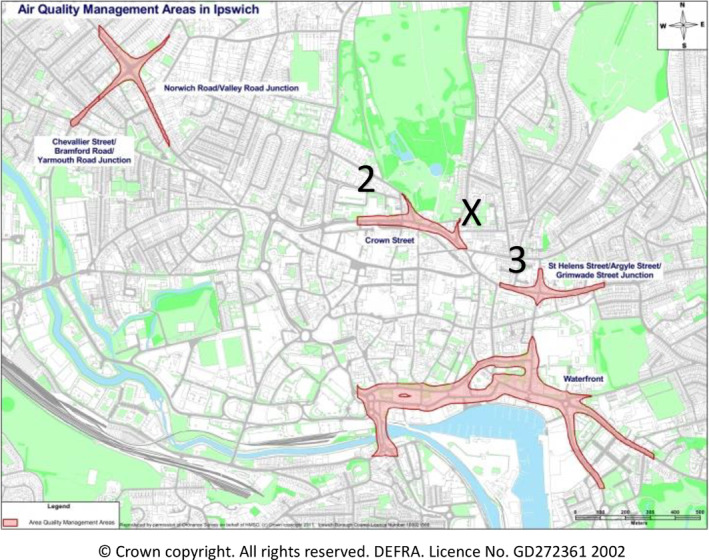
Air quality management areas (2) and (3) in Ipswich (Suffolk, UK), shaded in red, and the location of the primary school partner (X).

Both walking routes were in the centre of Ipswich, a town located in the county of Suffolk in the east of England. The town has a population of approximately 139,600 residents according to the most recent Census. The roadside route is located in Air Quality Management Areas 2 and 3 as defined by the local authority and the UK Air Information Resource (https://uk‐air.defra.gov.uk/aqma/local‐authorities?la_id=133; Figure 2). For context, the average annual nitrogen dioxide (NO_2_) levels along the roadside route are between approximately 25 and 42 μ g.m^3^ according to the local authority's 2022 Air Quality Annual Status Report (Ipswich Borough Council 2022), and the average daily traffic flow is approximately 13,500 vehicles (https://roadtraffic.dft.gov.uk/manualcountpoints/38524). The park is immediately adjacent to the school; there are no NO_2_ or other pollutant data for the park prior to the start of this study. The walking routes were designed so that distance covered and elevation change were similar. The school's catchment area for student admissions includes streets that are to the north, east, south and west of the park, so both the roadside and park walking routes are typical active travel journeys for students.

#### 2.1.2 Air Pollution

Fine (PM_2.5_) and coarse (PM_10_) particulate matter, along with volatile organic compounds (VOCs), were measured during all walks using four Plume Flow air sensors (PlumeLabs, Paris, France). Following laboratory chamber evaluation, these sensors have been recommended as useful for individuals or groups to monitor exposure to particulate matter pollution (Crnosija et al. 2022). The sensors were held at the children's approximate head height by members of the research team walking with the children. The same four Flow sensors were used for all walks to provide aggregate data as recommended (Crnosija et al. 2022).

#### 2.1.3 Fractional Exhaled Nitric Oxide

FeNO measurements were taken immediately before and after each walk at 15:20 h ± 5 min and 15:50 h ± 5 min, respectively, using a chemiluminescence analyser (NIOX VERO, Circassia, Sweden) to assess airway inflammation. All tests were conducted in accordance with the 2005 recommendations of the American Thoracic Society and the European Respiratory Society (American Thoracic Society and European Respiratory Society 2005). Children remained seated for the duration of the test and were instructed to maximally inhale with their lips sealed around the mouthpiece of the breathing handle to eliminate exogenous nitric oxide from the breath sample. Following this, children followed the device's visual instructions to maintain an expiratory flow rate of 50 mL/s for either 6 or 10 s test duration, which was determined by age and expiratory ability, which has been previously validated (Crater et al. 2017). Changes in children's FeNO have been observed immediately following exercise, returning to baseline within 30 min (Gabriele, Pijnenburg, Monti, Hop, Bakker, and De Jongste 2005a), so the single pre‐ to post‐walk measurements have a good rationale and precedent.

#### 2.1.4 Statistical Analysis

Test–retest reliability for all six pre‐walk FeNO measurements was assessed using mean rating (*k* = 6), absolute rating, 2‐way mixed effects model intraclass correlation coefficient (ICC) and their 95% confidence intervals. The mean of the three pre‐ and post‐walk FeNO values for each of the roadside and park routes were used to assess for a difference in absolute FeNO values for each walking route using a paired‐samples *t*‐test. Effect sizes were calculated and interpreted according to Cohen's *d*. Differences in FeNO change values (post minus pre values) for all three roadside and three park walks were assessed using repeated measures ANOVA with post hoc Bonferroni correction applied. The same test was used for measured air pollutants of PM_2.5_, PM_10_ and VOCs. Potential association between change in FeNO as the dependent variable and PM_2.5_, PM_10_ and VOCs were tested for with Pearson’s correlation coefficient. PM_2.5_, PM_10_ and VOCs were log‐transformed for analysis; change in FeNO was not log‐transformed given the prevalence and relevance of negative values.

All analyses used two‐sided tests, and *p*‐values less than 0.05 were considered statistically significant. ICC analysis was performed with IBM SPSS Statistics (version 27); paired‐samples *t*‐test, repeated measures ANOVA and Pearson’s correlation coefficient were conducted with GraphPad Prism (version 9).

## 3 Results

### 3.1 Walking Routes

**FIGURE 3:**
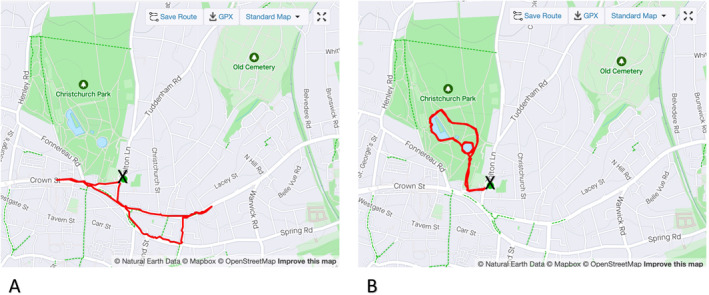
Roadside (A) and park (B) walking routes identified by red lines. The school location, the start and end point are identified (X). Credit to OpenStreetMap as displayed in the copyright notice, and data are available under the Open Database License.

The mean walking duration for the roadside condition was 30.1 ± 1.8 min of the total elapsed time and 27.0 ± 1.8 min of moving time; the difference caused by waiting at road crossings enroute. The mean walking duration for the park condition was 28.8 ± 1.2 min; there was no requirement to stop walking on the park route. The distances walked in that time were 2.08 ± 0.10 km for the roadside and 2.23 ± 0.05 km for the park route, with an elevation change of 22.9 ± 0.5 m and 27.5 ± 1.7 m, respectively. Examples of both walked routes are presented in Figure 3. There were no differences in walk duration or distance between roadside and park routes (*p* > 0.05 in all cases).

### 3.2 Air Pollution and Environmental Conditions

Measured air pollutants and other relevant meteorological conditions are presented in Table 1 for both roadside and park walking routes. There were no differences between any of the measured variables (*p* > 0.05 in all cases).

**TABLE 1:** Pollutant concentrations and meteorological variables (mean ± SD).

	Roadside route	Park route
PM_2.5_ (μg/m^3^)	5.93 ± 2.18	6.51 ± 2.56
PM_10_ (μg/m^3^)	14.91 ± 11.89	14.82 ± 8.12
VOCs (ppb)	133 ± 91	80 ± 50
Temperature (°C)	10.48 ± 3.50	9.35 ± 2.88
Wind speed (m.s^−1^)	6.61 ± 2.78	6.53 ± 2.22
Humidity (%)	76.2 ± 11.8	68.3 ± 9.5
Pressure (kPa)	100.5 ± 1.4	101.0 ± 1.2

### 3.3 Fractional Exhaled Nitric Oxide

For all pre‐walk measurements, the mean FeNO was 21.19 ± 17.34 ppb and the median was 15 ppb. Using the children specific thresholds from the ATS Clinical Practice Guidelines as a reference, 61% of the pre‐walk baseline measurements were in the < 20 ppb or “eosinophilic inflammation and responsiveness to corticosteroids are less likely” range, 22% were in the 20–35 ppb or “should be interpreted cautiously and with reference to the clinical context” range and 17% were in the > 35 ppb or “eosinophilic inflammation and, in symptomatic patients, responsiveness to corticosteroids are likely” range. All measurements that satisfied the test requirements previously mentioned were included for analysis.

**FIGURE 4:**
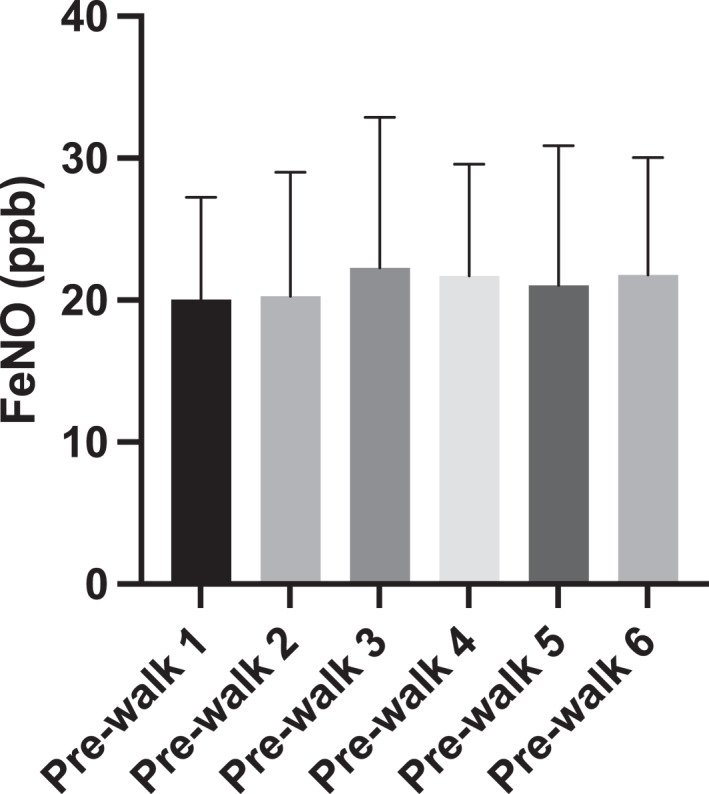
Group mean (with 95% CI) baseline and pre‐walk FeNO (ppb) values for all six walks.

**FIGURE 5:**
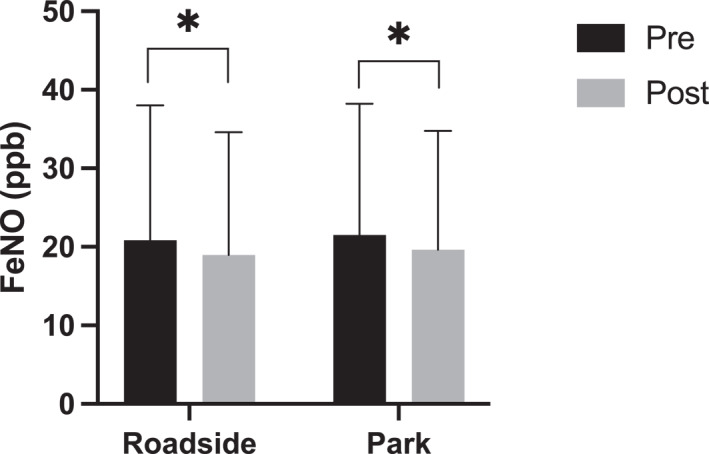
Significant reductions in pre‐walk *vs*. post‐walk FeNO for both roadside (*p* = 0.006) and park (*p* = 0.008) routes. Data are presented as group mean ± SD.

**FIGURE 6:**
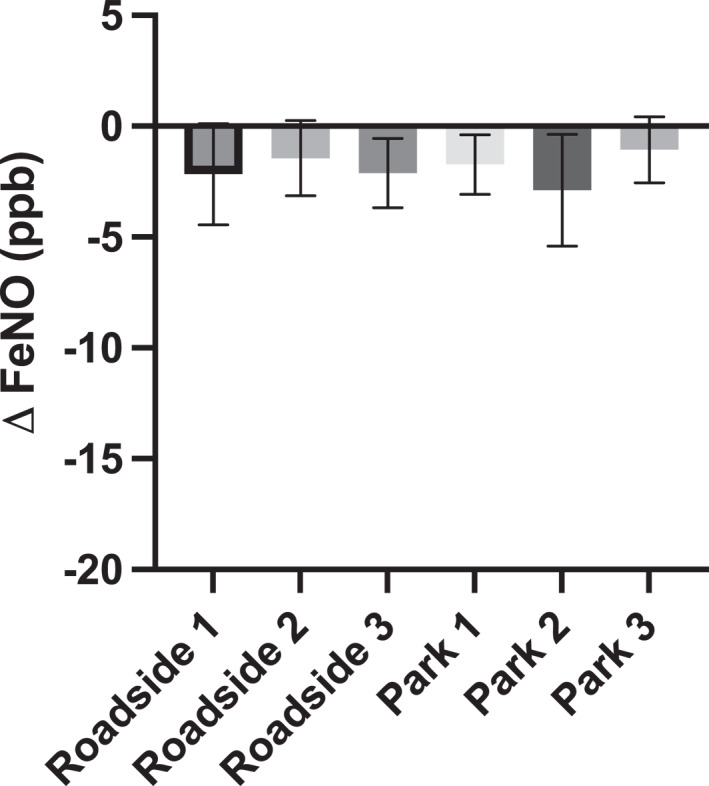
Group mean (with 95% CI) change in fractional exhaled nitric oxide (FeNO) for all three roadside and three park walking routes. There were no significant differences in FeNO change scores after each walk (*p* > 0.05).

The ICC for all baseline pre‐walk FeNO measurements was 0.882 (95% CI: 0.792, 0.947; Figure 4) classified as good. There was a reduction in FeNO values following walks in both roadside (20.87 ± 17.14 vs. 18.96 ± 15.63 ppb, *p* = 0.006 and *d* = 0.12) and park walking routes (21.52 ± 16.69 vs. 19.63 ± 15.15 ppb, *p* = 0.008 and *d* = 0.10) presented in Figure 5. The reduction was not significantly different between the walking routes (*p* > 0.05). There were no significant differences across any FeNO change scores between roadside (−2.17 ± 4.60, −1.44 ± 3.42 and −2.11 ± 3.14 ppb) and park walking routes (−1.72 ± 2.70, −2.89 ± 5.06 and −1.06 ± 3.00 ppb; Figure 6). There were no associations between change in FeNO and any of PM_2.5_ (*r* = −0.058 and *p* = 0.53), PM_10_ (*r* = −0.108 and *p* = 0.25) or VOCs (*r* = −0.126 and *p* = 0.17) pollutants measured during the walks. Given the lack of single model relationships, multiple regression was not required or appropriate as one of the key assumptions for multiple regression had been violated.

## 4 Discussion

This real‐world crossover study investigated the acute FeNO response to walking for 30 min along roadside and park routes after school in 18 children. There were no differences in pollution concentrations between roadside and park walking routes, and there was an equal reduction in FeNO following 30 min of walking in both environments. Changes in FeNO were not correlated with any pollutant concentrations. We also determined good FeNO reliability when the measurement is taken at the end of the school day. To the best of our knowledge, our study is one of very few if not the first before–after (pre‐post) study to measure and compare the effect of walking roadside and park routes on acute changes in FeNO in school children.

The reduction in FeNO compared to baseline values following 30 min of walking in both roadside and park locations were statistically significant, although trivial in terms of effect sizes. They do appear to be a typical response following physical activity in children without ambient air pollution being considered (Evjenth et al. 2013; Gabriele, Pijnenburg, Monti, Hop, Bakker, and De Jongste 2005b). For example, Evjenth and colleagues found FeNO to be reduced by 27.4% following running on a treadmill for 6–8 min compared to baseline values in non‐asthmatic children. Similarly, Gabriele and colleagues found a significant reduction in FeNO of 5 ppb 5 min post exercise following a six‐minute walking test in children with asthma (Gabriele, Pijnenburg, Monti, Hop, Bakker, and De Jongste 2005a). The reduction reported in our study, of approximately 2 ppb, or approximately 10% of pre‐walk values is modest compared to those mentioned, although the physical activity would be of lower intensity compared to Evjenth and colleagues. The physical activity in this study was a walk at approximately 4.5 km.hr^−1^, representing low to moderate intensity exercise. Further, the children participating in this study ordinarily walk to and from school and therefore will have been chronically exposed to ambient air pollution; this was not a new stimulus for these participants.

A strength of the current study was the use of personal air pollution sensors to measure ambient PM_2.5_, PM_10_ and VOCs, as opposed to fixed site monitoring stations, which typically underestimate commuter air pollution exposure (Martins et al. 2021). The method employed in this study allowed us to directly compare personal air pollution exposure in these two locations, which are different but close to one another. They are both viable walking routes to and from this school for the students that live within the recruitment catchment area. Parks are often suggested as an ideal alternative route for active travel to avoid busy roads where possible. However, pollution exposure in parks is rarely measured, especially when the location of the park is surrounded by busy roads. This study has shown that concentrations of PM2.5 and PM10 were similar in roadside and park locations where the locations are close to one another. It has been previously shown that particulate matter will disperse from its source with even light to moderate wind conditions (Cichowicz et al. 2017).

Given all pollutant concentrations were below current WHO daily thresholds, it is reasonable to expect FeNO will be reduced as we observed. Moreover, given there were no differences in the characteristics of the walking exercise or the pollutant concentrations between the roadside and park environments, it is also logical that both walking routes resulted in the same physiological response. In other words, although higher pollutant concentrations alone have been associated with increases in FeNO (Karakatsani et al. 2017), it is likely that the air pollution was not potent enough to influence the expected FeNO response to light physical activity. Furthermore, as none of the pollutants in single correlation models were correlated with the change in FeNO, it is likely that the physical activity stimulus alone is responsible for the decrease in FeNO following 30 min of walking in this study.

There is no clear consensus across the literature regarding the mechanisms responsible for acute exercise‐induced reductions in FeNO. One proposal is related to the increased oxygen utilisation and minute ventilation in response to exercise (Araneda et al. 2016), and that constitutive nitric oxide (NO) production in the pulmonary tissue will be partly determined by the oxygen concentration of the surrounding tissue. The increased oxygen utilisation during any physical activity will result in a decreased partial pressure of oxygen in the blood (Ortiz‐Prado et al. 2019) and this will be present when exercise stops. Because of its bronchodilatory and vasodilatory properties, there is a greater utilisation of NO in the pulmonary tissues and this greater utilisation of NO would naturally result in a decrease in the amount, which is exhaled (FeNO) immediately following exercise (Nosarev et al. 2015), as reported in this study. However, should an individual be exposed to higher levels of pollution than measured in this study, it is possible that increased FeNO following a bout of exercise will be found (Kubesch et al. 2015). This alternative finding would indicate an inflammatory response in the airways where inducible nitric oxide synthase forms of NO production are instead dominant (Luiking et al. 2010).

Although FeNO measurements have previously been reported to have good reproducibility in children (Kharitonov et al. 2003), to our knowledge, our study is the first to assess the reliability of FeNO measurements when taken on multiple separate occasions at the end of the school day in a real‐world setting. We found baseline FeNO to be reliable for children at the end of the school day. Our findings demonstrate that it is therefore feasible to conduct ecologically valid and reliable FeNO research with children in a real‐world setting. In this field of research, it is imperative that we are able to conduct research in a real‐world setting to capture and understand the implications of children's exposure to air pollution and the impact this may have on health parameters. Considering children spend a significant proportion of their day at school, it is important to consider the feasibility of conducting research around the school day to (i) uphold the validity and reliability requirements of any measured variables and (ii) create minimal disruption to the usual planned learning of a school day.

It is interesting to observe the variance in baseline FeNO across participants as represented by the large group standard deviation and range of low to high FeNO values recorded. This likely means recruitment bias was avoided. The within‐participant consistency was good as represented by the ICC outcome. Noteably, 39% of the children reliably presented baseline FeNO values that likely indicate some eosinophilic inflammation. This group of children presented higher resting FeNO than has been presented in the literature previously (Carlsen et al. 2016). With our data, it is not possible or appropriate to attempt to draw further conclusions about why this may be the case or how the school being situated in an Air Quality Management Area (defined by nitrogen dioxide levels) might influence this. However, it is undoubtedly of further interest for future research.

The pollution concentrations recorded in the current study do not exceed the daily guidelines for PM_10_ and PM_2.5_, but the recorded PM_2.5_ concentrations would exceed annual thresholds set by the WHO (World Health Organisation 2021). However, pollutants were only measured during data collection sessions (for 30 min on 22 separate days) and not for an annual period. Therefore, we cannot claim PM_2.5_ exceeded annual thresholds in the study locations. It is interesting for future research to consider the appropriateness of reporting pollutant concentration in relation to daily and annual thresholds when data are captured over more than one day but not for an entire year. Previous work has suggested that chronic exposure to low levels of pollution may have a more serious impact on people's health compared to sporadic exposure to higher concentrations of pollution (Moreno et al. 2003; Chen et al. 2008). Although the current study measured the impact of walking on FeNO in different environments on several occasions, the aim was still to assess the acute response to physical activity. It will be beneficial for future studies to measure the effect of different routes for active travel on health measures over a longer period and with appropriate control this can include a broader age range and therefore stages of lung development. As the real‐world studies progress, work should focus on clinically vulnerable groups (e.g., children with long‐term cardio and/or respiratory conditions in particular).

Regarding limitations, our study aimed to measure nitrogen dioxide. However, there were too many missing data for the results to be valid, and therefore nitrogen dioxide concentrations are not reported. The study was conducted during a time of the year when deciduous trees and plants would be dormant and without leaves, although this of course does represent real‐world conditions for a significant portion of the calendar year, so highly relevant and ecologically valid. Trees can ‘trap’ or absorb airborne particulate matter and potentially provide some protection against human inhalation (Freer‐Smith et al. 2005; Tomašević et al. 2005), although it is not a guarantee, and more can be read in this adjacent area of environmental science (Kumar et al. 2019). Simply, we are not claiming that tree canopies were a factor in this study. Lastly, future studies should aim to monitor FeNO in the hours after exercise to observe any potential lag effects.

In summary, this study found that FeNO was reduced in school children following 30 min walks in both roadside and park environments, and that the roadside and park environments resulted in similar levels of reduced FeNO. Although we recognise the relatively small participant numbers, it is possible to suggest that walking both roadside and park location creates a physiological response that is indicative of a positive response to exercise. Importantly, this has been observed on this occasion where pollutant concentrations do not exceed the daily 2021 WHO Air Quality Guideline thresholds. We may also conclude that it is possible to measure FeNO reliably in children at their school location, supporting the practice of real‐world observation or intervention research in this discipline.

## Ethics Statement

The study was approved by the University of Suffolk Research Ethics Committee (ref: RETH(S)22/027).

## Conflicts of Interest

The authors declare no conflicts of interest.

## Data Availability

The data that support the findings of this study are available from the corresponding author upon reasonable request.
